# Detection of Aflatoxigenic Fungi in Dairy Cattle Feed: Findings From Urban Districts of Tigray, Ethiopia

**DOI:** 10.1002/vms3.70362

**Published:** 2025-05-05

**Authors:** Sisay Weldegebriel Zeweld, Enquebaher Kassaye Tarekegn, Meressa Abraha Welearegay

**Affiliations:** ^1^ Department of Veterinary Public Health and Food Safety Mekelle University College of Veterinary Sciences Mekelle Ethiopia; ^2^ Department of Chemistry Mekelle University College of Natural and Computational Sciences Mekelle Ethiopia

**Keywords:** aflatoxigenic fungi, ammonia vapour test, dairy feed, fungal load count, UV light test

## Abstract

The growing Ethiopian dairy sector faces significant challenges, including feed contamination by aflatoxigenic fungi, limiting productivity and endangering public health. A cross‐sectional survey was conducted from August to November 2024 to investigate the prevalence of aflatoxigenic fungi in dairy cattle feed sampled from 143 farms across Mekelle, Wukro, Adigrat, Korem and Alamata, as well as the associated risk factors in urban districts of Tigray, Ethiopia. Feed samples, including hay, straw, wheat/maize middling and commercial concentrates, were analysed for fungal contamination using UV light, ammonia vapour tests and colony counts (colony‐forming units per gram [CFU/g]). The analysis revealed that 33.6% of samples contained aflatoxigenic fungi (*Aspergillus flavus* and *Aspergillus parasiticus*), whereas 66.4% were contaminated with non‐aflatoxigenic species. Average fungal loads were 2.11 × 10^5^ CFU/g, with dairy farms in Korem town showing the highest contamination (2.495 × 10^5^ CFU/g) and dairy farms in Mekelle city the lowest (1.839 × 10^5^ CFU/g) (*F* = 125.15, *p* = 0.001, *η* ^2^ = 0.784). Feed moisture content (*F* (1, 135) = 4.041, *p* = 0.046), environmental conditions (26°C–27°C, >60% humidity) and poor storage practices (*F* (1, 135) = 223.65, *p* = 0.001) significantly increased contamination levels. Storage on soil floors led to higher fungal loads (*F* = 223.65, *p* = 0.001, *η* ^2^ = 0.613), whereas owner awareness of aflatoxins reduced contamination (*F* = 78.27, *p* = 0.001, *η* ^2^ = 0.357). The findings highlight the importance of enhancing feed storage practices, providing training to farmers and developing collaborative mitigation strategies. These efforts are crucial for reducing aflatoxin contamination, improving dairy productivity and safeguarding public health.

## Graphical abstract

This study investigates aflatoxigenic fungi in dairy cattle feed from urban districts of Tigray, Ethiopia, using culture, microscopy, UV fluorescence and ammonia vapour tests. *Aspergillus flavus* and *A. parasiticus* are detected in 33.6% of samples. Key risk factors include high humidity, poor storage and low farmer awareness. Improved storage and training are essential to reduce contamination and enhance dairy productivity and safety.

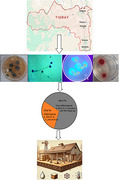

## 1 Introduction

The Ethiopian dairy sector is growing due to rising demand for dairy products, offering income opportunities for smallholder farmers (Shapiro et al. 2017; Weldegiorgis et al. 2019; Park and Chemere 2023). However, dairy farming faces significant challenges, including feed scarcity, climate change and fluctuating rainfall patterns. These challenges are particularly pronounced in urban and peri‐urban areas, which rely heavily on purchased feeds (Moje et al. 2023). The high cost of commercial feed puts financial pressure on smallholder farmers, making it difficult for them to sustain their production. This is compounded by poor nutrition, inadequate management, disease and the lack of proper breeding programmes, all of which contribute to low dairy cow productivity (Korir et al. 2023; Nyokabi et al. 2023; Tenagne et al. 2023). Feed shortages and health issues, such as fungal contamination, significantly affect dairy productivity. Cereal grains used for cattle feed are at risk of contamination by mycotoxins, particularly aflatoxins, which can cause liver damage and immune suppression (Atungulu and Mohammadi‐Shad 2019). In Ethiopia, dairy cattle feeds like forage, cut‐and‐carry pasture, wheat/maize middling, wheat‐bran and atella (a by‐product of traditional home‐brewed beer, which is currently replacing commercial concentrates) pose a high risk of aflatoxigenic fungi contamination during production and storage (Worku et al. 2015). Studies show that up to 78% of concentrate feed exceeds safe aflatoxin levels (Jonathan et al. 2024; Martins et al. 2024). Dairy feed contamination by fungi, such as *Aspergillus* spp., *Penicillium* spp., *Fusarium* spp. and others, is common (Elgarhi et al. 2020; Saber et al. 2022). Aflatoxigenic fungi, notably *Aspergillus flavus* and *Aspergillus parasiticus*, produce aflatoxins that contaminate dairy feed (Awuchi et al. 2021; Elkenany and Awad 2021).

In addition to the challenges of limited feed availability, the use of contaminated feed, particularly cereal grains and roughage infected with aflatoxigenic fungal species, introduces a significant risk to dairy production. Therefore, effective detection methods for aflatoxigenic fungi are essential (Gebremariam and Belay 2016). Conventional and molecular techniques are commonly used for fungal detection in dairy feeds (Acharya and Hare 2022; Abd El‐Aziz et al. 2021; Pisoschi et al. 2023). Conventional methods involve culture‐based approaches, using agar media like Sabouraud dextrose agar (SDA) and potato dextrose agar (PDA) to isolate and enumerate fungal colonies (Geethalakshmi et al. 2021). Koch's standard method and microscopic examination of fungal morphology also aid in species identification, relying on colony colour, texture and spore arrangement (Variane et al. 2018; Rostam 2021). *Aspergillus* and *Penicillium* are commonly isolated from dairy feeds, with *Aspergillus* species like *A. flavus*, *Aspergillus niger* and *A. parasiticus* exhibiting distinct colony colours and conidiophore structures (Elgarhi et al. 2020; Tesfaye et al. 2024; Adelusi et al. 2022). High fungal contamination rates, ranging from 18.84 × 10^3^ to 55.23 × 10^3^ colony‐forming units per gram [CFU/g], are found in dairy feeds in Ethiopia, with seasonal variations influencing fungal diversity, especially for *Aspergillus* and *Fusarium* species during warmer months (Saber et al. 2022; Tesfaye et al. 2024).

Morphological characteristics under microscopic examination, such as conidiophore structure and conidia arrangement, help in identification (Elgarhi et al. 2020). *A. flavus* and *A. parasiticus* are identified based on their macroscopic and microscopic features on PDA. *A. flavus* colonies appear yellowish‐green to green and velvety and grow 5–8 cm in diameter within 5–7 days at 25°C, with compact, radiate conidial heads. *A. parasiticus* colonies are light yellow to yellow‐green, with looser conidial arrangements and a faster growth rate of 6–10 cm in the same period (Elgarhi et al. 2020; Tesfaye et al. 2024; Adelusi et al. 2022). Microscopically, *A. flavus* has smooth, unbranched conidiophores, small round vesicles and globose conidia in chains. *A. parasiticus* exhibits similar conidiophores but larger vesicles and more uniform conidia. Differences in colony colour, conidial arrangement and growth rate distinguish the two species (Saber et al. 2022; Kandasamy et al. 2019). Key microscopic features, including spore morphology and hyphal septation, further aid in differentiation (Okayo et al. 2020). Additionally, fluorescence under UV light and colour changes in the presence of chemicals like ammonium hydroxide help in identifying toxin‐producing strains (Lebar et al. 2018; Akbar et al. 2023).

Ammonium hydroxide solution (28%) is a significant advancement in mycotoxin management, offering a rapid and cost‐effective method for detecting aflatoxigenic fungi. The ammonia vapour test is inexpensive, reliable and fast, making it ideal for pre‐screening contaminated samples in agricultural settings. This method relies on the alkaline properties of ammonium hydroxide to induce colour changes in fungal colonies. When exposed to ammonia vapours, *A. flavus* colonies develop a reddish‐pink hue, whereas *A. parasiticus* colonies turn pink. These colour changes occur as ammonia reacts with fungal metabolites like kojic acid, intermediates in aflatoxin biosynthesis (Wang et al. 2022; Sowmya 2022; Poungpong et al. 2024). The simplicity and speed of ammonium hydroxide screening make it a practical alternative to labour‐intensive methods like chromatography, with results available within 15–30 min. It is particularly valuable in resource‐limited settings where rapid screening is essential. Studies highlight its high sensitivity to aflatoxin‐producing strains, making it an effective diagnostic tool for food and feed safety (Sowmya 2022). This method is also useful in conjunction with other detection strategies like the dichlorvos–ammonia method. Additionally, ammonia treatment has been shown to reduce aflatoxin levels in contaminated food products, contributing to aflatoxin mitigation (Yabe et al. 2015; Kushiro et al. 2018). In practical terms, agar plates inoculated with test samples are incubated, and colony characteristics are observed, including growth rate, pigmentation and exudate production. Microscopic examination and biochemical tests then confirm species identity and assess aflatoxigenicity (Salisu et al. 2020; Senanayake et al. 2020). These microbiological detection methods are crucial in assessing aflatoxin contamination, particularly for safeguarding dairy cattle feed and maintaining the integrity of the dairy industry in the Tigray region of Ethiopia.

The Tigray region of Ethiopia has experienced significant disruptions in agricultural production, leading to food and feed shortages. Limited access to quality feed has particularly affected dairy farming in urban areas. Inadequate storage conditions, including prolonged exposure of stored grains to moisture and humidity, have created favourable environments for mould growth, increasing the risk of aflatoxin contamination in animal feed. Despite the urgency of this issue, no previous studies have been conducted in Tigray to detect aflatoxigenic fungal species in dairy cattle feed. This research gap hindered efforts to safeguard animal and human health from aflatoxin exposure. This study, the first of its kind in Tigray, aimed to fill this critical knowledge gap. Through cultural methods, UV light and ammonia vapour tests, the research sought to isolate and identify aflatoxigenic fungal species in dairy cattle feed and to assess the potential risk factors contributing to fungal growth. By identifying the prevalence of aflatoxin‐producing fungi, the study aimed to enhance the safety and sustainability of animal feed, contributing to the ongoing recovery process of the region. The findings from this research are intended to inform future interventions to mitigate aflatoxin contamination, thereby improving food security, animal health and public safety in Tigray.

## 2 Materials and Methods

### 2.1 Study Area

The study was conducted in urban districts of Tigray, Ethiopia, including Mekelle, Wukro, Adigrat, Korem and Alamata, chosen for their expanding dairy sectors and active farmer participation. These areas were categorized on the basis of environmental factors as highland (Mekelle, Korem and Adigrat, 2000–2700 m above sea level) and middle altitude (Alamata and Wukro, 1500–2000 m above sea level). Data from the December 2023 report of the Tigray Bureau of Agriculture and Rural Development indicate a total of 227 dairy farms in these areas: 74 in Mekelle, 32 in Wukro, 53 in Adigrat, 25 in Korem and 43 in Alamata.

### 2.2 Research Design

A cross‐sectional study was conducted from August to November 2024 to isolate and identify aflatoxigenic fungal species in dairy cattle feed samples from selected dairy farms in the urban districts of Tigray.

### 2.3 Sampling Technique and Sample Size Determination

Five urban towns, Alamata, Korem, Mekelle, Wukro and Adigrat, were purposely selected due to their potential for dairy farming. Dairy farms with five or more milking cows were included in the study. The sample size was determined using the Thrusfield (2018) formulas for an infinite population, with a 95% confidence interval, 5% precision and 50% prevalence. The initial sample size was approximately 384.16, which was adjusted for the finite population size of 227 to an adjusted sample size of 143. The farms were selected proportionally and randomly, with Mekelle contributing 47 farms, Wukro 20, Adigrat 33, Korem 16 and Alamata 27. Feed samples representing common feed types (hay, straw, wheat/maize middling and commercial concentrates) were collected and analysed for total fungal counts and aflatoxigenic fungal species.

### 2.4 Study Methods

#### 2.4.1 Isolation and Identification of Aflatoxigenic Fungi in Dairy Feed

A total of 23 samples were collected from commercial concentrates, 49 from hay, 33 from straw and 38 from wheat/maize middling and bran among the 143 dairy farms included in the study. In this study, commercial concentrates refer to feed mixtures made from ground grains (such as wheat, maize, oats, sorghum and barley) and oilseed by‐products. On the other hand, roughages in this study refer to fibrous plant materials such as hay, straw, wheat/maize bran or grain by‐products, cut‐and‐carry pasture and atella. Dairy feed samples (100 g) were collected using proper handling techniques and stored in sterile containers. Fifty grams were sealed in plastic bags for moisture content determination, whereas the remaining 50 g were kept in an icebox and transported to the laboratory for fungal analysis. Aflatoxigenic fungal species were isolated in the Microbiology Laboratory at Mekelle University using both macroscopic and microscopic morphological characteristics. Feed samples were processed using peptone water (0.1%) for serial dilution (up to 10^6^) and cultured (volume plated = 0.1 mL) on SDA plates, incubated at 27°C for 7 days (Greco et al. 2014). The SDA and PDA plates were prepared with chloramphenicol (100 mg/L) to prevent bacterial growth. After 24 h of incubation of the plates at 27°C, samples were inoculated with the sample and incubated for 7 days. Fungal colonies resembling *A. flavus* and *A. parasiticus* were selected for sub‐culturing on PDA plates for further characterization. The identification of *A. flavus* and *A. parasiticus* was based on their macroscopic and microscopic characteristics. *A. flavus* colonies appeared yellowish‐green to green with compact, radiate conidial heads, whereas *A. parasiticus* colonies were light yellow‐green with looser conidial arrangements and a faster growth rate (Elgarhi et al. 2020; Tesfaye et al. 2024; Adelusi et al. 2022). Microscopic identification involved examining conidiophores, vesicles and conidial arrangement. *A. flavus* had smooth, unbranched conidiophores, small round vesicles and globose conidia in chains. *A. parasiticus* exhibited larger vesicles, sub‐globose to oval conidia and a more dispersed arrangement (Saber et al. 2022; Kandasamy et al. 2019). Key microscopic features, including spore morphology and hyphal septation, further aided differentiation (Okayo et al. 2020).

Fungal load was quantified by counting colonies on SDA plates after 7 days of incubation. Results were recorded as CFU/g of feed using the plate count method (Greco et al. 2014). Lactophenol cotton blue staining was used for microscopic examination to identify key morphological traits, including conidiophores and vesicles, aiding in the identification of *Aspergillus* species (Elgarhi et al. 2020; Adelusi et al. 2022). After isolating *A. flavus* and *A. parasiticus* on PDA, colonies were transferred to fresh PDA plates and slants and incubated at 27°C for 7 days. Fluorescence under UV light and ammonia vapour tests were performed to confirm aflatoxigenicity. *A. flavus* exhibited a greenish‐yellow fluorescence, whereas *A. parasiticus* displayed blue–green fluorescence. The ammonia test showed characteristic colour changes in the colonies, helping classify them as aflatoxigenic (Arifah et al. 2022; Sukmawati et al. 2018).

#### 2.4.2 Fungal Enumeration

Fungal enumeration was conducted using the colony counting method. Serially diluted samples were plated on PDA and incubated at 27°C for 7 days. Colonies were manually counted, and fungal load was expressed as CFU/g using the standard formula. Counts from plates with 30–300 colonies were recorded, and final CFU values were calculated as mean ± standard deviation from triplicates. AFPA was not used for fungal or aflatoxigenic species detection due to its unavailability in the local Ethiopian market. Instead, PDA media and PDA agar slants were used for fungal isolation, whereas UV light fluorescence and ammonia vapour tests were applied to detect aflatoxigenic species.

#### 2.4.3 Determination of Moisture Content of Dairy Feed

Moisture content was determined using the oven drying method, where feed samples were dried at 105°C until a constant weight was achieved. Moisture content was calculated using the difference in weight before and after drying (Association of Official Analitycal Chemist (AOAC) 2005).

#### 2.4.4 Relative Humidity (RH) of Storage Areas

The RH in dairy feed storage areas was measured using a digital hygrometer. Measurements were recorded at different times within the same day, with RH categorized as ideal (50%–60%), high (>65%) and low (<40%) (Koteswara Rao et al. 2016).

#### 2.4.5 Questionnaire Survey

A structured questionnaire was developed specifically for this study to collect data on factors influencing fungal contamination and aflatoxigenic species growth in dairy feed. The English‐language version of the questionnaire has been provided as Supplementary File. The survey included categorical independent variables, such as farm location, farm owner's gender, education status, dairy management training, farm altitude, knowledge of mould/aflatoxin, dairy feed type, grazing system, feed storage period, feed type and storage conditions. The dependent variables include the total fungal colony count and nature and type of the fungal species detected (aflatoxigenicity), as they are influenced by various independent factors. Additionally, continuous covariates such as the average temperature of the study area, feed storage area temperature (°C) and feed moisture content (%) were assessed for their potential impact on fungal growth.

### 2.5 Data Quality Assurance and Statistical Analysis

Data validation was performed by triplicate testing of samples. Standard scientific protocols and laboratory procedures ensured accuracy, minimizing potential errors. The study analysed categorical variables (e.g., farm location, feed type) and continuous covariates (e.g., temperature, moisture content). Descriptive statistics and regression analyses (binary, multinomial logistic regression, ANCOVA) were used to examine associations between variables and fungal contamination, with statistical significance set at *p* ≤ 0.05.

## 3 Results

### 3.1 Fungal Isolation and Identification in Dairy Feed Samples

A total of 143 feed samples were collected from 5 study sites—Adigrat, Alamata, Korem, Mekelle and Wukro. Of the roughage feeds, hay was the most common feed type (34.3%), followed by wheat/maize middlings (26.6%) and straw (23.1%), whereas commercial concentrates accounted for 16.1%. Fungal contamination was widespread, with 33.6% of samples testing positive for aflatoxigenic species (*A. flavus* and *A. parasiticus*) and 66.4% contaminated with non‐aflatoxigenic fungi, including non‐aflatoxin‐producing *A. flavus* and *A. parasiticus, Aspergillus fumigatus*, *A. niger*, *Aspergillus terreus*, *Penicillium* spp., *Fusarium* spp., *Rhizopus* spp. and *Mucor* spp. Although non‐aflatoxigenic fungi do not produce aflatoxins, their presence indicated the complexity of fungal contamination in the dairy feeds (Table 1).

**TABLE 1:** Distribution of fungal species detected in feed samples from different study areas.

The type of sample taken for analysis	Study areas					Total	
	Mekelle	Wukro	Adigrat	Korem	Alamata		
Commercial concentrates	8	5	3	4	3	23 (16%)	
Hay	16	5	13	7	8	49 (34.3%)	
Straw	12	5	6	4	6	33 (23.1%)	
Wheat/maize middling	11	5	11	1	10	38 (26.6%)	
Total	47	20	33	16	27	143 (100%)	

Type of the fungal species detected						Prevalence	
*Aspergillus flavus* only	4	1	3	2	3	13 (9.1%)	
*Aspergillus parasiticus* only	2	1	1	1	2	7 (4.9%)	48 (33.6%)
Both *A. flavus* and *A. parasiticus*	7	3	5	6	7	28 (19.6%)	
Non‐aflatoxigenic *A. flavus* and *A. parasiticus*	9	4	5	2	4	24 (16.8%)	95 (66.4%)
Other fungal species	25	11	19	5	11	71 (49.6%)	
Total	47	20	33	16	27	143	

**FIGURE 1:**
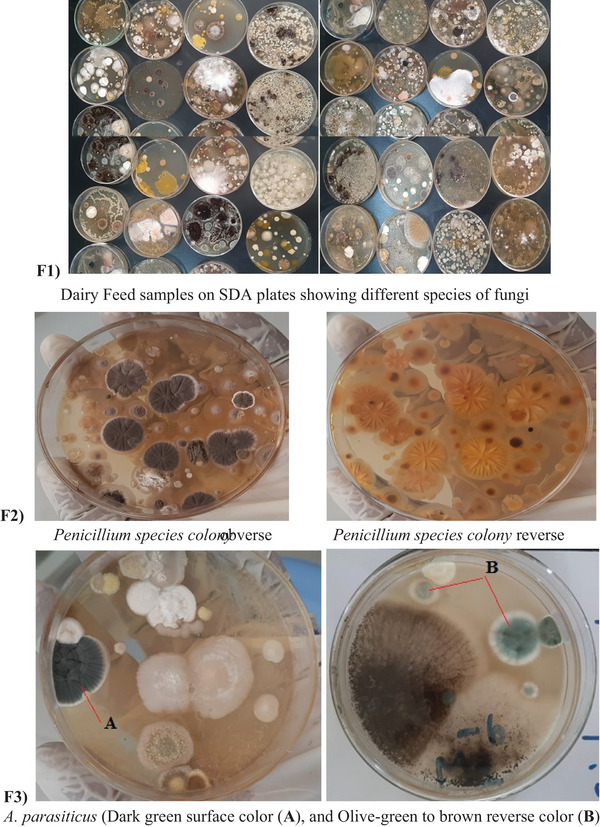
Macroscopic morphological characteristics of fungal colonies isolated from dairy feed samples cultured on SDA plates (F1–F3): (F1) Dairy feed samples on SDA plates showing different species of fungi; (F2) *Penicillium* species colony obverse and *Penicillium* species colony reverse; (F3) *Aspergillus parasiticus* (dark green surface colour (A) and olive‐green to brown reverse colour (B)).

**FIGURE 2:**
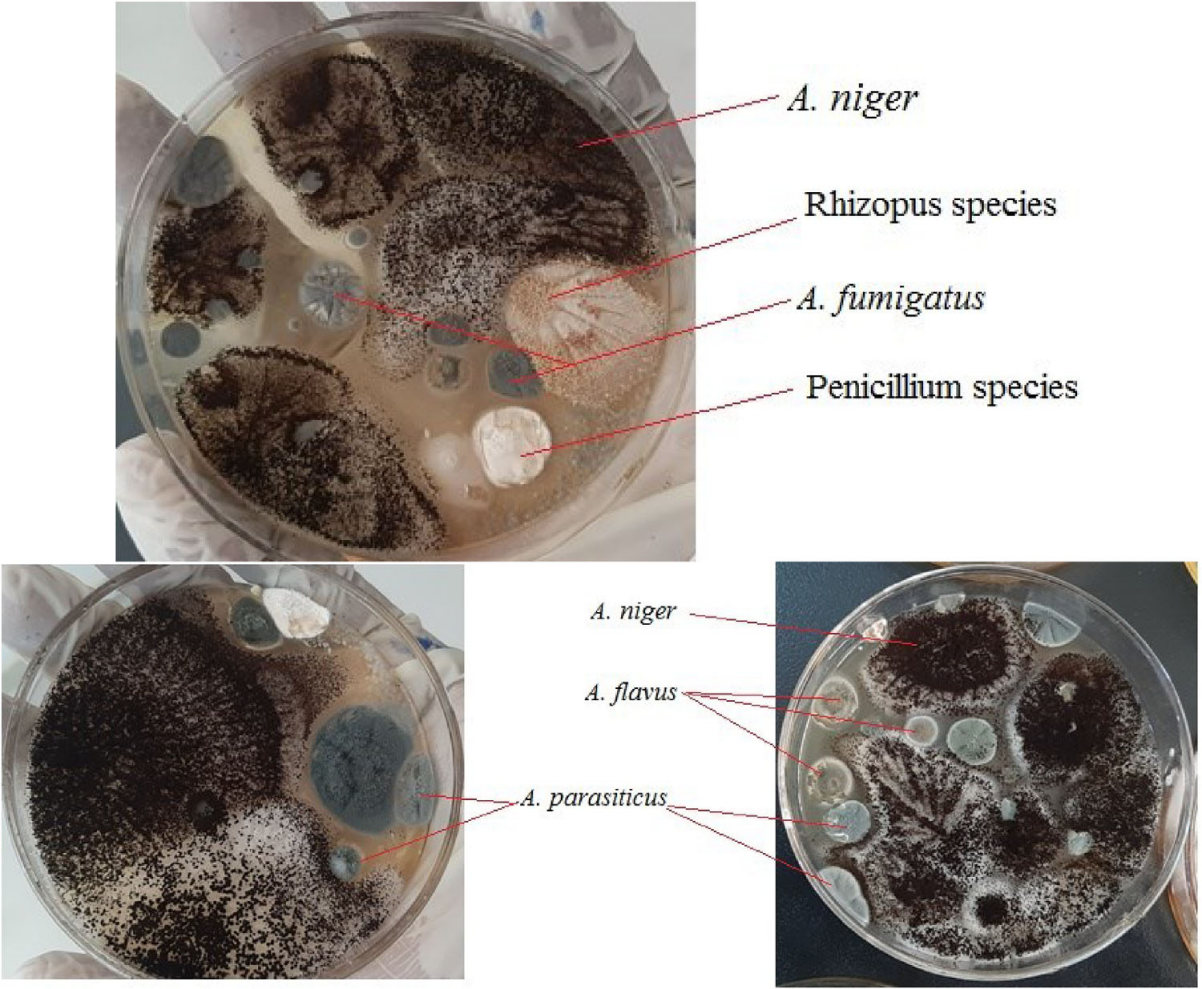
Types of fungal colonies identified on the basis of macroscopic morphological characteristics on SDA plates.

**FIGURE 3:**
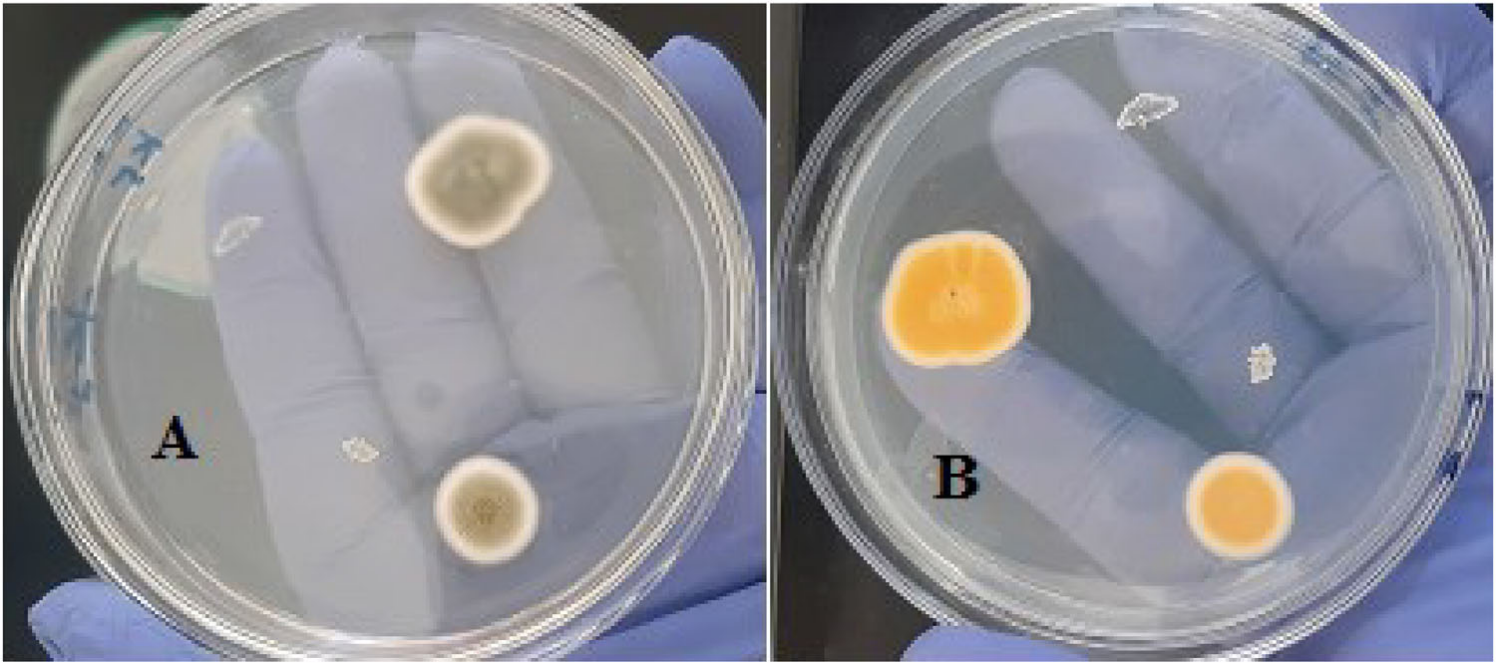
*Aspergillus flavus* (yellow‐green surface side colour (A) and pale‐brown to yellowish or golden reverse side colour (B) on PDA plate).

The macroscopic and microscopic characteristics of fungal colonies isolated from dairy feed samples were described, including their colony morphology on SDA and PDA plates, which showed colour variations in *A. flavus* and *A. parasiticus* (Figures 1, 2, 3).

**FIGURE 4:**
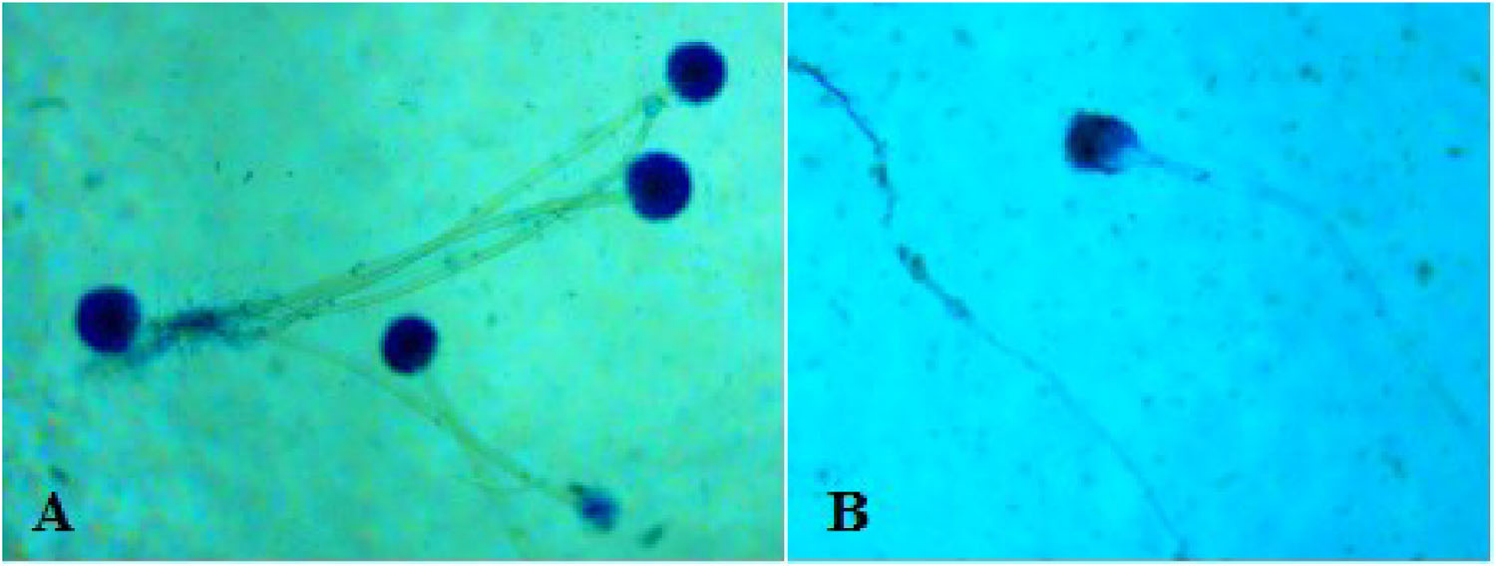
Microscopic appearances of *Aspergillus flavus* (A) with globose or spherical conidial heads and *Aspergillus parasiticus* (B) with oval conidial heads, showing conidiophore and conidial head structures, stained with lactophenol cotton blue (100×).

The microscopic structures, including conidiophores and conidial heads, were observed under 100× magnification after staining with lactophenol cotton blue, revealing globose conidial heads in *A. flavus* and oval conidial heads in *A. parasiticus* (Figure 4).

### 3.2 Fungal Colony Count Comparison and Variability Analysis

Fungal colony counts were compared between two feed types—‘Roughage’ (grain by‐products, cut‐and‐carry pasture and atella) and ‘Commercial concentrates’. There was no significant difference in fungal colony count variability between the groups (*F* (1, 141) = 0.322, *p* = 0.571). The mean colony counts for both groups were similar (2.11 ×  10^5^ and 2.12 × 10^5^ CFU/g, respectively). The mean fungal colony count varied slightly among sample types, with commercial concentrates having the lowest (2.05 × 10^5^), followed by hay (2.13 × 10^5^), straw (2.12 × 10^5^) and wheat/maize middling (2.13 × 10^5^). Levene's test showed no significant differences in variability across sample types (*F* (3, 139) = 0.693, *p* = 0.558), indicating the sample type did not significantly affect fungal colony counts. The overall mean fungal colony count was 2.12 × 10^5^ CFU/g (Table 2).

**TABLE 2:** Descriptive statistics, Levene's test for equality of variances and fungal colony count variation based on dairy feed type and sample taken.

Type of feed	*N*	Mean	SD	Levene's test (1, 141)	
				*F*	*p*
Roughage (grain by‐products, cut‐and‐carry pasture and atella)	116	2.11 × 10^5^	2.69 × 10^4^	0.322	0.571
Commercial concentrates	27	2.12 × 10^5^	2.76 × 10^4^		
Total	143	2.12 × 10^5^	2.70 × 10^4^		

Type of sample taken	*N*	Mean	SD	Levene's test (3, 139)	
				*F*	*p*
Commercial concentrates	23	2.05 × 10^5^	2.92 × 10^4^	0.693	0.558
Hay	49	2.13 × 10^5^	2.76 × 10^4^		
Straw	33	2.12 × 10^5^	2.82 × 10^4^		
Wheat/Maize middling	38	2.13 × 10^5^	2.41 × 10^4^		
Total	143	2.12 × 10^5^	2.70 × 10^4^		

The ‘Tests of Between‐Subjects Effects’ revealed that the model explained only a small portion of the variance in fungal colony counts (*R* ^2^ = 0.079). The intercept had a significant effect on fungal counts (*F* (1, 135) = 77.204, *p* < 0.001, *η* ^2^ = 0.364). However, the sample type did not significantly influence fungal contamination (*F* (3, 135) = 0.138, *p* = 0.937, *η* ^2^ = 0.003). Moisture content significantly affected fungal contamination (*F* (1, 135) = 4.041, *p* = 0.046, *η* ^2^ = 0.029), with higher moisture levels increasing fungal growth. The interaction between sample type and moisture was not significant (*F* (3, 135) = 0.062, *p* = 0.980, *η* ^2^ = 0.001).

Descriptive statistics of moisture content categories and corresponding fungal colony counts are shown in Table 3. Higher moisture levels (above 12%) were associated with increased fungal colony counts, suggesting that moisture content is a key factor influencing fungal contamination in feed. Pair‐wise comparisons showed no significant differences in fungal counts across sample types (*p* > 0.05), further supporting the conclusion that moisture content, rather than sample type, plays a significant role in fungal growth.

**TABLE 3:** Analysis of variance (ANOVA) for factors influencing fungal colony count: sample type, moisture of feed and their interaction.

Source	Type III sum of squares	df	Mean square	*F*	Sig	Partial eta squared (*η* ^2^)
Corrected model	8.15 × 10^9^	7	1.16 × 10^9^	1.651	0.126	0.079^*^
Intercept	5.44 × 10^10^	1	5.44 × 10^9^	77.204	0.000	0.364
Sample type	2.92 × 10^8^	3	9.72 × 10^7^	0.138	0.937	0.003
Moisture of feed	2.85 × 10^9^	1	2.85 × 10^9^	4.041	0.046	0.029
Sample type^*^ moisture of feed	1.31 × 10^8^	3	4.35 × 10^7^	0.062	0.980	0.001
Error	9.52 × 10^10^	135	7.05 × 10^8^			
Total	6.50 × 10^12^	143				
Corrected total	1.03 × 10^11^	142				

* *R* squared = 0.079 (adjusted *R* squared = 0.031).

### 3.3 Analysis of Fungal Colony Counts Across Different Study Areas and Influencing Factors

The analysis revealed significant variations in fungal colony counts across study areas. Dairy farms in Mekelle city had the lowest mean (1.839 × 10^5^ CFU/g), whereas Korem town had the highest (2.495 × 10^5^ CFU/g). The differences were statistically significant (*F* = 125.15, *p* = 0.001), explaining 78.4% of the variance (*η* ^2^ = 0.784). Temperature also influenced fungal growth, with higher counts at 26°C and 27°C compared to 18°C and 22°C (*F* = 125.15, *p* = 0.001, *η* ^2^ = 0.784). RH in feed storage significantly affected fungal growth, with higher RH (>60%) leading to a mean of 2.231 × 10^5^ CFU/g, compared to 1.795 × 10^5^ CFU/g at 50%–60% RH (*F* = 149.36, *p* = 0.001, *η* ^2^ = 0.514). Floor type also played a role, with soil floors supporting higher colony counts (2.268 × 10^5^ CFU/g) compared to concrete (1.824 × 10^5^ CFU/g) (*F* = 223.65, *p* = 0.001, *η* ^2^ = 0.613). Dairy farm owners’ knowledge about mould or aflatoxin significantly reduced fungal contamination, with aware farms showing a mean of 1.775 × 10^5^ CFU/g compared to 2.191 × 10^5^ CFU/g in unaware farms (*F* = 78.27, *p* = 0.001, *η* ^2^ = 0.357). These findings highlight the importance of environmental conditions and farm management practices in fungal contamination (Table 4).

**TABLE 4:** Analysis of variance (ANOVA) results for factors influencing fungal colony counts (CFU/g).

Factor	Mean (CFU/g)	*N*	SD	*F*	*p*	*Eta squared* (*η* ^2^)
Study areas				125.15	0.001	0.784
Mekelle	1.839 × 10^5^	47	1.208 × 10^4^			
Wukro	1.966 × 10^5^	20	8.880 × 10^3^			
Adigrat	2.238 × 10^5^	33	1.461 × 10^4^			
Korem	2.495 × 10^5^	16	1.068 × 10^4^			
Alamata	2.332 × 10^5^	27	1.464 × 10^4^			
Environmental temperature				125.15	0.001	0.784
18°C	2.495 × 10^5^	16	1.068 × 10^4^			
22°C	2.238 × 10^5^	33	1.461 × 10^4^			
25°C	1.839 × 10^5^	47	1.208 × 10^4^			
26°C	1.966 × 10^5^	20	8.880 × 10^3^			
27°C	2.332 × 10^5^	27	1.464 × 10^4^			
RH% of the feed storage compartment				149.36	0.001	0.514
Ideal RH (50%–60%)	1.795 × 10^5^	38	9.783 × 10^3^			
High RH (>60%)	2.231 × 10^5^	105	2.175 × 10^4^			
Type of floor of storage area				223.65	0.001	0.613
Soil floor	2.268 × 10^5^	94	1.939 × 10^4^			
Concrete floor	1.824 × 10^5^	49	1.018 × 10^4^			
Knowledge of dairy farm owners about mould or aflatoxin				78.27	0.001	0.357
Yes	1.775 × 10^5^	26	1.158 × 10^4^			
No	2.191 × 10^5^	117	2.332 × 10^4^			

The ANCOVA analysis showed that the overall model was significant (*F* (8, 134) = 64.71, *p* = 0.001), explaining 79.4% of the variance in fungal colony counts (*R* ^2^ = 0.794). The geographical location had the most significant impact on fungal contamination (*F* (4, 134) = 45.15, *p* = 0.001, *η* ^2^ = 0.574), whereas sample type (*F* (3, 134) = 1.22, *p* = 0.306, *η* ^2^ = 0.027) and temperature (*F* (1, 134) = 2.61, *p* = 0.109, *η* ^2^ = 0.019) did not significantly affect fungal growth. The study highlights the key role of geographic factors in fungal contamination, with minimal influence from sample type or storage conditions (Table 5).

**TABLE 5:** Results of between‐subjects effects analysis on factors influencing total fungal colony count across different sample types and study areas.

Source	Type III sum of squares	df	Mean square	*F*	*Sig*	Partial eta squared (*η* ^2^)
Corrected model	8.21 × 10^7^ ^a^	8	1.03 × 10^7^	64.71	0.001	0.794
Intercept	2.69 × 10^7^	1	2.69 × 10^7^	169.97	0.001	0.559
Sample type	5.79 × 10^8^	3	1.93 × 10^8^	1.22	0.306	0.027
Study area	2.86 × 10^7^	4	7.16 × 10^6^	45.15	0.001	0.574
Temperature of feed storage area	4.13 × 10^8^	1	4.13 × 10^8^	2.61	0.109	0.019
Error	2.12 × 10^7^	134	1.59 × 10^6^			
Total	6.50 × 10^12^	143				
Corrected total	1.03 × 10^11^	142				

^a^ *R* squared = 0.794 (adjusted *R* squared = 0.782).

### 3.4 Prevalence of Aflatoxigenic Species: UV Light and Ammonia Vapour Screening

**FIGURE 5:**
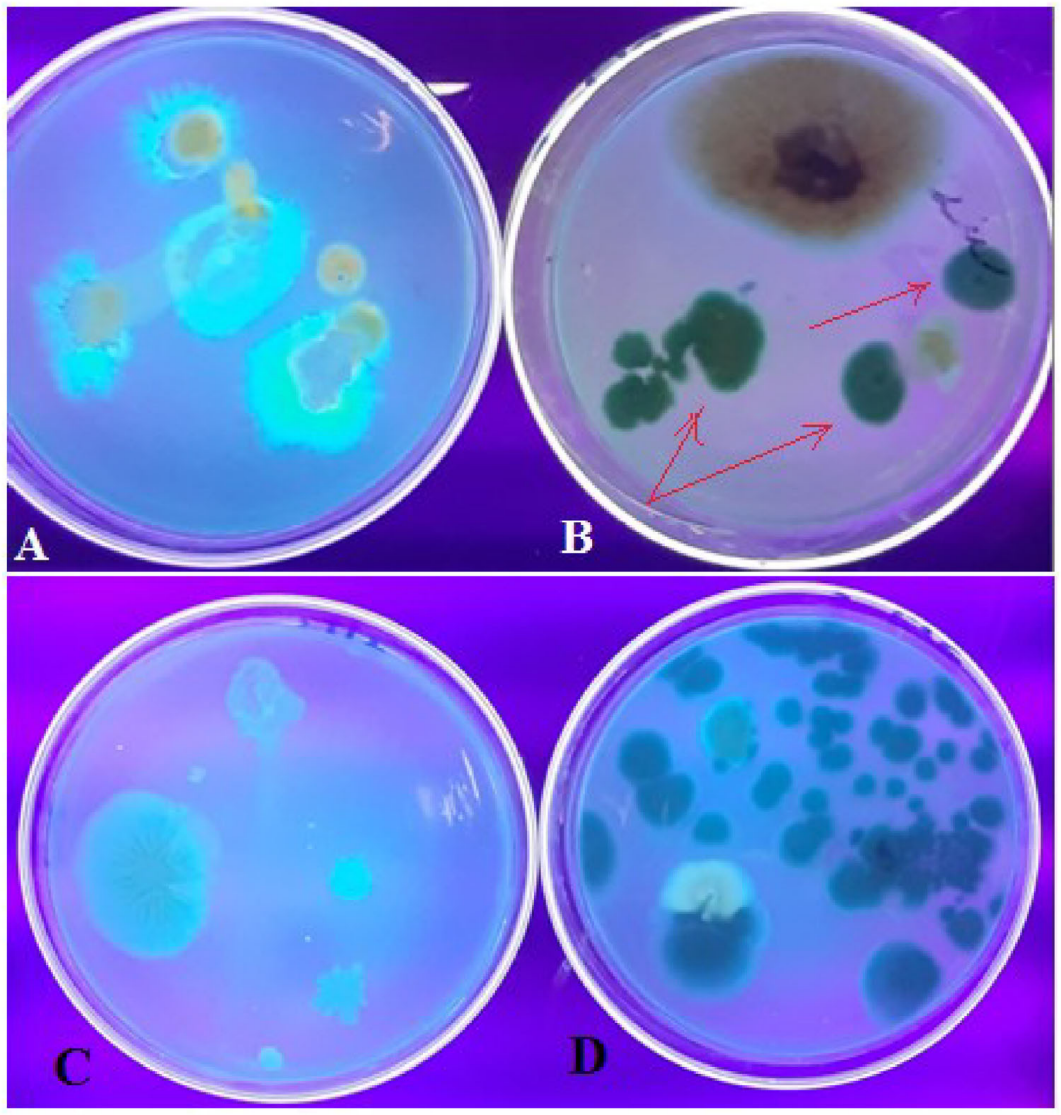
Aflatoxigenic species of *Aspergillus flavus* (A, blue) and *Aspergillus parasiticus* (B, green fluorescence) on PDA plates. *A. flavus* predominantly produces aflatoxin B types (B1 and B2), which only fluoresce blue (C), and *A. parasiticus* typically produces a wider range of aflatoxins, including both B (blue‐fluorescing) and G (green‐fluorescing) types (D).

**FIGURE 6:**
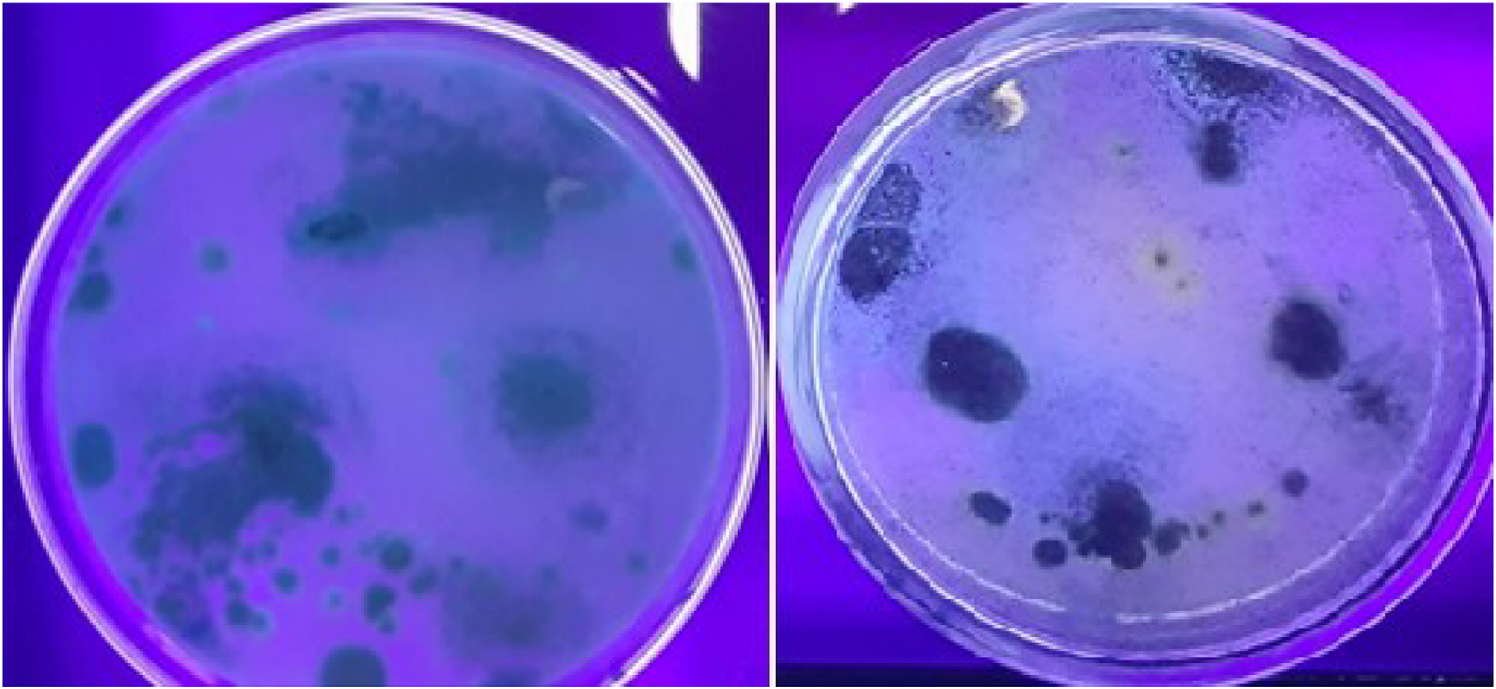
Non‐aflatoxigenic species of *Aspergillus flavus* (left) and *Aspergillus parasiticus* (right) with no distinct colour of UV fluorescence.

**FIGURE 7:**
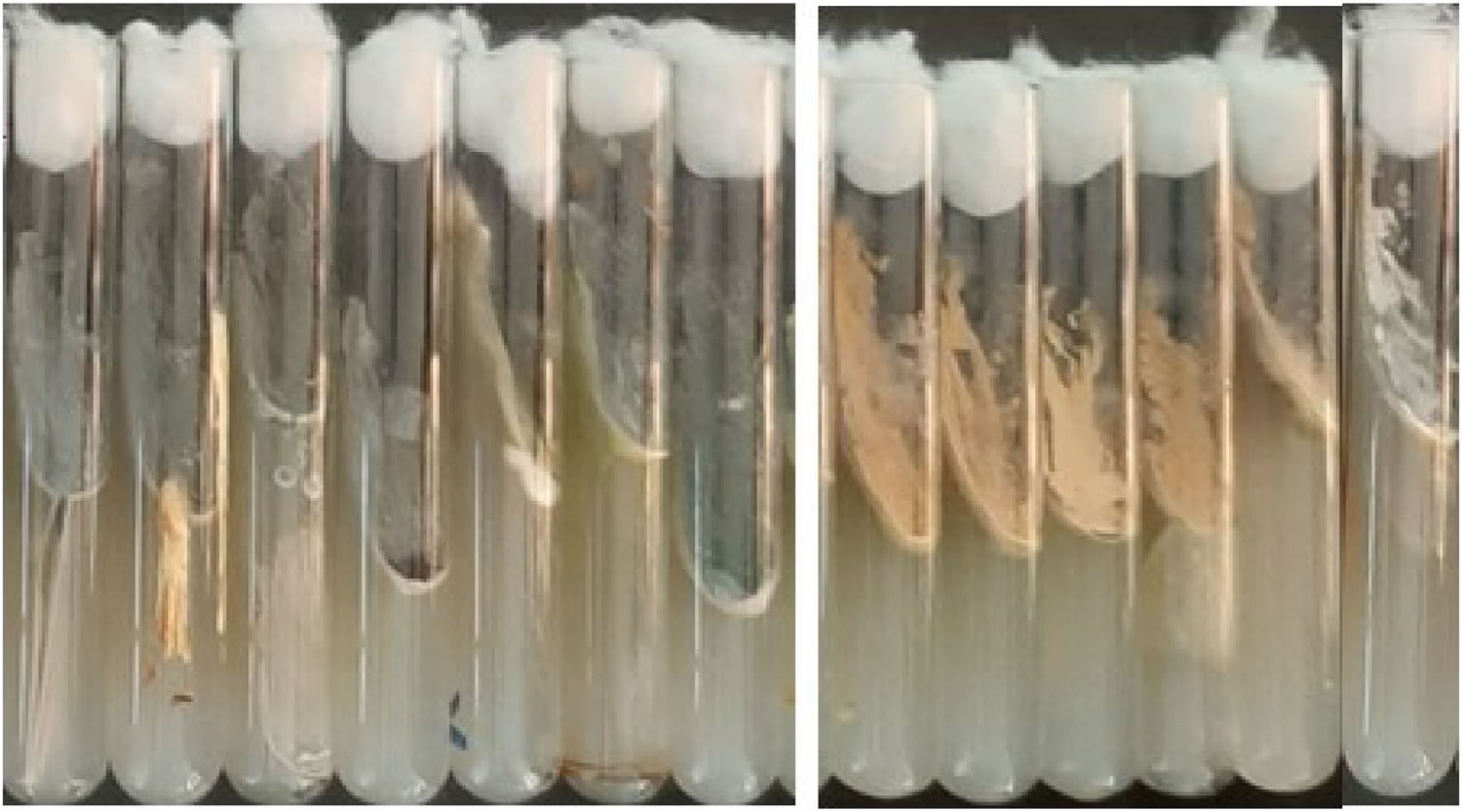
Pure colonies of *Aspergillus parasiticus* (left) and *Aspergillus flavus* (right) on PDA slants.

**FIGURE 8:**
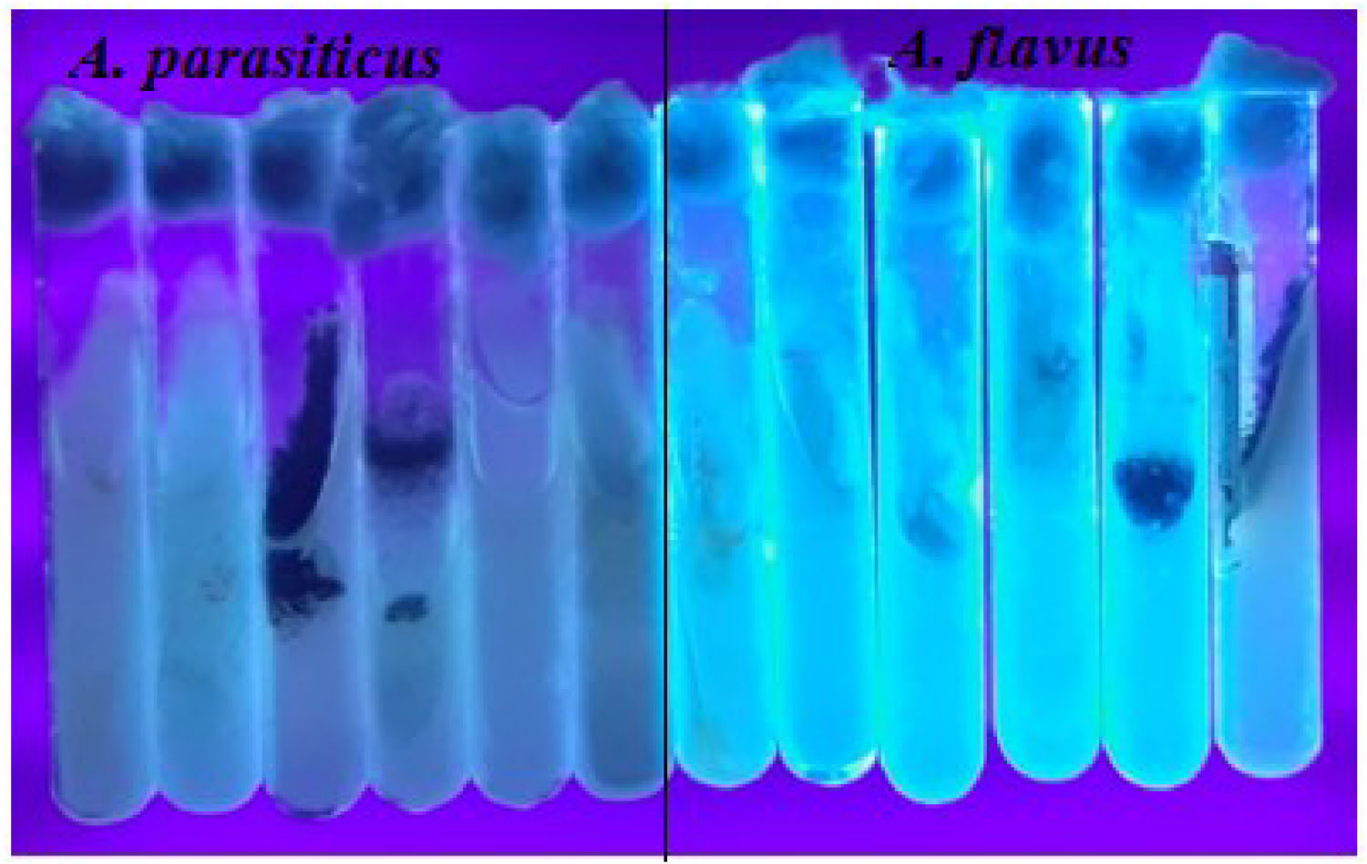
Aflatoxigenic *Aspergillus parasiticus* (light green) and *Aspergillus flavus* (bluish) UV light fluorescence on PDA slants.

**FIGURE 9:**
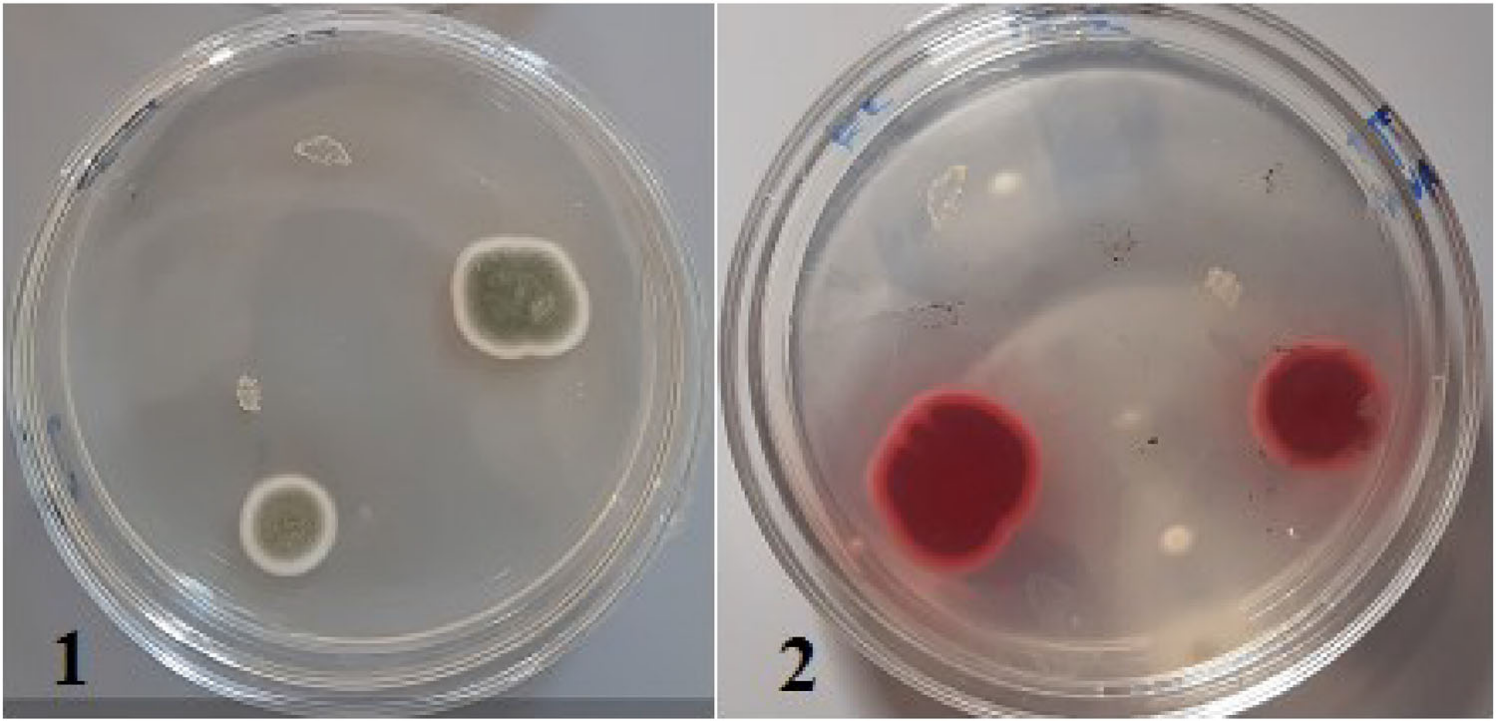
Confirmation of aflatoxigenicity of *Aspergillus flavus* using ammonia vapour test: (1) initial observation and (2) development of dark red or pink colour after 30 min of exposure to ammonium hydroxide solution.

This study used two methods, UV light fluorescence and the ammonia vapour test, to detect aflatoxigenic species. All positive samples exhibited characteristic blue and green fluorescence under UV light, confirming their aflatoxigenic potential. The ammonia vapour test further confirmed this, producing yellow and orange colours. Both methods were consistent in identifying aflatoxigenic species. The study also compared fungal colony counts between aflatoxigenic and non‐aflatoxigenic species, with UV fluorescence patterns and colony morphology on PDA media, as well as ammonia vapour test results, confirming the differentiation (Figures 5, 6, 7, 8, 9).

Aflatoxigenic species had a higher mean fungal colony count (*M* = 2.30 × 10^5^; SD = 24,860.84) compared to non‐aflatoxigenic species (*M* = 2.02 × 10^5^; SD = 23,162.17). The overall mean fungal colony count was 2.12 × 10^5^ (SD = 26,974.95). Levene's test showed no significant difference in variances between the groups (*F* (1, 141) = 1.352, *p* = 0.247). A univariate analysis of variance (ANOVA) explained 27.5% of the variance (*R* ^2^ = 0.275, adjusted *R* ^2^ = 0.264). Significant effects were found for fungal species type (*F* (1, 140) = 41.347, *p* = 0.001, *η* ^2^ = 0.228) and moisture content (*F* (1, 140) = 8.492, *p* = 0.004, *η* ^2^ = 0.057) (Table 6).

**TABLE 6:** Total fungal colony count by aflatoxigenicity and feed moisture content: Levene's Test, descriptive statistics and analysis of variance (ANOVA) results.

Nature of the detected fungal species	Mean	SD	*N*	Levene's test *F*	Levene's test Sig.
Aflatoxigenic species	2.30 × 10^5^	24860.84	48	1.352	0.247
Non‐aflatoxigenic *Aspergillus flavus, Aspergillus parasiticus* and other species	2.02 × 10^5^	23162.17	95		
Total	2.12 × 10^5^	26974.95	143		

Source	Type III sum of squares	df	Mean square	*F*	*Sig*	Partial eta squared
Corrected model	2.84 × 10^10^ ^a^	2	1.42 × 10^10^	26.524	0.001	0.275
Intercept	1.99 × 10^11^	1	1.99 × 10^11^	371.651	0.001	0.726
Aflatoxigenicity	2.21 × 10^9^	1	2.21 × 10^9^	41.347	0.001	0.228
Moisture content of feed	4.55 × 10^9^	1	4.55 × 10^9^	8.492	0.004	0.057
Error	7.49 × 10^10^	140	5.35 × 10^8^			
Total	6.50 × 10^12^	143				
Corrected total	1.03 × 10^11^	142				

^a^ *R* squared = 0.275 (adjusted *R* squared = 0.264).

Of the 72 samples testing positive for *A. flavus* and *A. parasiticus*, 48 (33.6%) were aflatoxigenic. In Adigrat, 42.42% of the samples tested positive for *A. flavus* and *A. parasiticus*, whereas 57.58% were other species. In dairy farms of Alamata town, 59.26% were aflatoxigenic, and in Korem, 68.75% tested positive for *A. flavus* and *A. parasiticus*. The highest co‐detection rates occurred in Korem (38%) and Alamata (26%), showing a widespread presence of aflatoxigenic species, posing a risk for aflatoxin contamination (Table 7).

**TABLE 7:** Prevalence and distribution of aflatoxigenic species across study areas.

Study sites	No. of samples	Positive *for Aspergillus flavus* and *Aspergillus parasiticus*	Other species	Aflatoxigenic species		
				*A. flavus*	*A. parasiticus*	Both
Adigrat	33	14 (42.42%)	19 (57.58%)	3 (9%)	1 (3%)	5 (15%)
Alamata	27	16 (59.26%)	11 (40.74%)	3 (11%)	2 (7%)	7 (26%)
Korem	16	11 (68.75%)	5 (31.25%)	2 (13%)	1 (6%)	6 (38%)
Mekelle	47	22 (46.81%)	25 (53.19%)	4 (9%)	2 (4%)	7 (15%)
Wukro	20	9 (45.00%)	11 (55.00%)	1 (5%)	1 (5%)	3 (15%)
Total	143	72 (50.35%)	71 (49.65%)	13 (9.1%)	7 (4.9%)	28 (19.6%)

### 3.5 Predictors of Aflatoxigenic Fungal Species Presence in Dairy Farms

The analysis identified significant associations between certain categorical variables and the presence of aflatoxigenic species. The study in dairy farms of Korem town revealed a significant relationship between geographical location and fungal contamination (*χ* ^2^ = 4.16, *p* = 0.041). Feed storage duration also influenced fungal presence; storage for 3–6 months (*χ* ^2^ = 11.511, *p* = 0.001) and over 6 months showed strong associations with contamination. However, fungal species type, environmental temperature, educational status, sample type and grazing system were not statistically significant (*p* > 0.05) (Table 8).

**TABLE 8:** Chi‐square test results for factors associated with the presence of aflatoxigenic species.

Variable	Category	Observed frequency	*χ* ^2^	*p* value
Type of fungal species detected	*Aspergillus flavus* only	13	—	—
	*Aspergillus parasiticus* only	7	—	—
	Both *A. flavus* and *A. parasiticus*	28	—	—
	Non‐aflatoxigenic *A. flavus* and *A. parasiticus*	24	—	—
	Other fungal species	71	—	—
Environmental temperature	18°C	16	—	—
	22°C	33	—	—
	25°C	47	—	—
	26°C	20	—	—
	27°C	27	—	—
Study area	Mekelle city	47	7.104	0.130
	Wukro town	20	0.765	0.382
	Adigrat town	33	0.762	0.383
	Korem town	16	4.16	0.041
	Alamata town	27	1.766	0.184
Educational status	Graduate	20	0.583	0.445
	Secondary school	29	0.305	0.581
	Primary school	58	0.001	0.973
	Illiterate	36	—	—
Sample type	Commercial concentrates	23	—	—
	Hay	49	0.907	0.341
	Straw	33	0.205	0.651
	Wheat/maize middling	38	0.249	0.618
Feed storage period	Less than 3 months	25	—	—
	3–6 months	36	11.511	0.001
	Greater than 6 months	82	—	—
Grazing system	Zero‐grazing	100	1.016	0.313
	Open grazing	13	0.705	0.401
	Semi‐intensive grazing	30	—	—

### 3.6 Result of Questionnaire Survey

The questionnaire administered to dairy farm owners across five study areas revealed significant findings about dairy farming practices. A notable gender disparity was observed, with 88.1% (*n* = 126) of farm owners being male and only 11.9% (*n* = 17) female. Geographically, Mekelle city had the highest representation (32.9%, *n* = 47), followed by Adigrat town (23.1%, *n* = 33), Alamata (18.9%, *n* = 27), Wukro (14%, *n* = 20) and Korem (11.2%, *n* = 16). Educationally, 40.6% (*n* = 58) of respondents had completed primary school, 25.2% (*n* = 36) were illiterate, 20.3% (*n* = 29) were secondary school graduates and 14% (*n* = 20) had higher education. Training in dairy management was limited, with only 27.3% (*n* = 39) having received training, whereas 72.7% (*n* = 104) had not.

Most farm owners (81.1%, *n* = 116) used locally available feed, such as roughage, grain by‐products and atella, whereas 18.9% (*n* = 27) supplemented with commercial concentrates. Feed storage practices were concerning, as 65.7% (*n* = 94) used soil floors, which may increase contamination risks, compared to 34.3% (*n* = 49) who had concrete floors. Knowledge about mould and aflatoxin contamination was low, with only 18.2% (*n* = 26) of farm owners aware of the risks, whereas 81.8% (*n* = 117) were unaware. Feed was mainly stored outdoors (68.5%, *n* = 98), with only 31.5% (*n* = 45) stored indoors, further exposing it to contamination risks. Zero‐grazing was the predominant system (69.9%, *n* = 100), followed by semi‐intensive grazing (21%, *n* = 30) and open grazing (9.1%, *n* = 13). Feed storage duration was also a concern, as 57.3% (*n* = 82) stored feed for more than 6 months, 25.2% (*n* = 36) for 3–6 months and 17.5% (*n* = 25) for less than 3 months. These findings indicate challenges in gender equity, education, training and feed management, which impact the safety and productivity of dairy farming (Table 9).

**TABLE 9:** Demographic and farm management characteristics of dairy farm owners.

Variable	Category	Frequency	Per cent
Gender of farm owner	Male	126	88.1
	Female	17	11.9
Study areas	Mekelle city	47	32.9
	Wukro town	20	14.0
	Adigrat town	33	23.1
	Korem town	16	11.2
	Alamata town	27	18.9
Educational status of dairy farm owners	Graduate	20	14.0
	Secondary school	29	20.3
	Primary school	58	40.6
	Illiterate	36	25.2
Previous training on dairy farm management	Yes	39	27.3
	No	104	72.7
The type of feed routinely provided to dairy cows	Roughage, grain by‐products, cut and carry pasture, atella	116	81.1
	Commercial concentrates	27	18.9
Type of floor of storage area	Soil floor	94	65.7
	Concrete floor	49	34.3
Knowledge of dairy farm owners about mould or aflatoxin	Yes	26	18.2
	No	117	81.8
Nature of feed storage environment	Indoor storage	45	31.5
	Outdoor storage	98	68.5
Type of grazing system	Zero‐grazing	100	69.9
	Open grazing	13	9.1
	Semi‐intensive grazing	30	21.0
Length of feed storage period	Less than 3 months	25	17.5
	3–6 months	36	25.2
	Greater than 6 months	82	57.3

## 4 Discussion

The study identified a widespread presence of *A. flavus* and *A. parasiticus* in dairy feed samples from five urban locations in Tigray: Adigrat, Alamata, Korem, Mekelle and Wukro. A total of 143 feed samples were analysed, with 50.4% testing positive for these species. Of the positive samples, 33.6% were found to be aflatoxigenic, whereas 16.8% were non‐aflatoxigenic. This indicates a significant risk of aflatoxin contamination, which was likely exacerbated by the scarcity of animal feed during and after the conflict in Tigray. The scarcity forced farmers to rely on poorly stored grain and long‐stored feed, which, under prolonged exposure to moisture and humidity, promoted mould growth and increased the prevalence of aflatoxigenic species. These findings are consistent with studies by Tesfaye et al. (2024), Tadesse et al. (2024), Motbaynor et al. (2021) and Sewunet et al. (2024), which reported similar fungal species in livestock feeds across Ethiopia.

When comparing our findings with previous studies, we observed a similar prevalence of *A. flavus* and *A. parasiticus* in dairy feeds. For instance, Tesfaye et al. (2024) found *A. flavus* to be the most common aflatoxigenic species in Ethiopian feeds, a result which aligns with our findings. Our study, like those of Khalifa et al. (2022) in Egypt and Adelusi et al. (2022, 2023) in South Africa, also found non‐aflatoxigenic strains of *A. flavus* and *A. parasiticus*. The findings across various regions underscore the widespread presence of these fungi in animal feed, posing significant health risks.

Regarding the detection methods, our study employed UV light fluorescence and ammonium hydroxide vapour tests, which effectively identified aflatoxigenic species. Previous studies, such as those by Sewunet et al. (2024), Egbuta et al. (2016) and Njobeh et al. (2010), also used these methodologies, reporting high contamination rates in maize and concentrate mixtures. In our study, these methods demonstrated high sensitivity and specificity in detecting aflatoxigenic fungi. However, although both methods proved effective, their limitations include the inability to quantify aflatoxin concentrations directly, which could be addressed by incorporating advanced techniques like PCR or ELISA, as demonstrated by Lagat et al. (2020) and Ortega et al. (2020).

In the context of fungal colony counts across different feed types, our study found no significant difference between ‘roughage’ (grain by‐products, cut‐and‐carry pasture and atella) and ‘commercial concentrates’ feeds, with counts of 2.11 × 10^5^ CFU/g and 2.12 × 10^5^ CFU/g, respectively. However, we did observe that commercial concentrates had slightly lower fungal counts than other feed types, consistent with Saber et al. (2022) in Algeria, who attributed this difference to better storage practices and lower moisture content. These results are in line with other studies, which have shown that fungal contamination is more closely related to environmental and storage conditions than to the type of feed (Sara and Ahmed 2023; Gnezdilova et al. 2024).

Furthermore, the study revealed significant variation in fungal colony counts across different dairy farm locations, with Korem showing the highest fungal count (2.495 × 10^5^ CFU/g) and Mekelle the lowest (1.839 × 10 CFU/g). These findings corroborate the results of Tesfaye et al. (2024) in eastern Ethiopia, who also found significant fungal contamination, with *A. flavus* being the most prevalent species. Environmental factors, such as temperature and humidity, played a critical role in fungal growth, with higher temperatures (26°C–27°C) and humidity levels (>60%) accounting for the majority of variation in fungal colony counts. This is consistent with the findings of Tesfaye et al. (2024) in South Africa. Moreover, storage conditions were a crucial factor; farms with soil floors had higher contamination levels compared to those with concrete floors, aligning with the results of Tadele et al. (2023) in the South Gondar Zone. Additionally, farms with informed owners had lower contamination levels, further emphasizing the importance of owner knowledge in managing feed contamination, a finding also reported by Gizachew et al. (2016).

Regarding the prevalence of aflatoxigenic fungi, our study found that *A. flavus* and *A. parasiticus* were the most common species, which is consistent with Tesfaye et al. (2024), who reported that *A. flavus* accounted for 80% of the *Aspergillus* species found in livestock feeds. The effectiveness of UV light fluorescence and ammonia vapour tests in detecting these species was confirmed by studies like those of Lakshman and Bellibatlu (2024) and Sowmya (2022), who also found these methods to be effective in detecting aflatoxigenic fungi.

Significant associations were observed between the location of the study area and the presence of aflatoxigenic species (*χ* ^2^ = 4.16, *p* = 0.041), as well as between feed storage duration and fungal contamination. Longer storage periods (3–6 months) were more prone to fungal growth, consistent with previous studies in Ethiopia and Africa that linked poor storage practices to higher contamination levels (Tesfaye et al. 2024; Casu et al. 2024; Nji et al. 2022; Boni et al. 2021; Birgen et al. 2020; Mutegi et al. 2018). Additionally, the study revealed a gender disparity in dairy farm ownership, with 88.1% of owners being male, which is consistent with the findings of other studies in Ethiopia and Africa, where male dominance in agriculture is common (Getaneh and Gebremedhin 2017; Galmessa et al. 2019; Somano 2014; Tefera 2020). However, the study also highlighted the important role of women in dairy management, as noted by Tadesse et al. (2020).

The study also found that 65.7% of dairy farm owners stored feed on soil floors, increasing the risk of contamination and mould growth. This is in line with previous studies in Ethiopia and Africa, which highlighted the importance of proper storage facilities in preventing fungal contamination (Tesfaye et al. 2024; Birgen et al. 2020). Additionally, 57.3% of farm owners stored feed for over 6 months, which further increased contamination risks, a trend also observed in Kenya and Ethiopia (Tesfaye et al. 2024; Birgen et al. 2020; Mutegi et al. 2018). Although 69.9% of farms practised zero‐grazing, which allows for more control over feeding, no significant differences in fungal contamination were observed across different farm management systems. This highlights the critical importance of effective feed storage practices in reducing contamination risks, as noted in previous studies (Debas et al. 2020; Nyabinwa et al. 2020).

Lastly, the study found a high prevalence of *A. flavus* and *A. parasiticus* in dairy feed, with fungal colony counts ranging from 2.5 × 10^4^ to 5.2 × 10^5^ CFU/g. This suggests a significant risk of aflatoxin contamination, which could lead to aflatoxin M1 contamination in milk. Given the established regulatory limits for AFM1 in milk (0.5 µg/kg by the FDA and 0.05 µg/L by the European Commission) (Yakubu and Vyas 2020; FDA 2020; European Commission Regulation (EC) 2006; van Egmond et al. 2006), further research is needed to assess aflatoxin levels in both feed and milk. Additionally, mitigation strategies, such as improved feed storage conditions and the application of antifungal treatments, should be explored to reduce the risks associated with aflatoxin contamination.

## 5 Conclusion and Recommendations

The study found significant contamination of dairy feed samples from Tigray, Ethiopia, with aflatoxigenic fungi, *A. flavus* and *A. parasiticus*. The contamination levels varied by geographical location, feed storage practices and climatic conditions. In particular, areas with higher humidity and inadequate feed storage practices experienced the highest levels of fungal contamination, highlighting the critical role of storage conditions in fungal growth and aflatoxin production. The findings emphasized the importance of proper feed storage in preventing fungal contamination, with moisture‐resistant containers, proper ventilation and airtight storage systems being essential to mitigate the risks. The study also examined the effectiveness of UV fluorescence and ammonia vapour tests for detecting these fungi. Although these tests were useful in identifying fungal contamination, the study did not include a gold standard method for comparison, and the sensitivity of these methods was not determined. Therefore, claims regarding the high sensitivity and reliability of these methods for early detection cannot be conclusively drawn from the results.

Given the significant presence of aflatoxin‐producing fungi in dairy feeds, which pose risks to livestock health and milk production, there is an urgent need for targeted interventions. To address this, it is essential to improve feed storage conditions, especially in high‐humidity areas. Farmers should be encouraged to use moisture‐resistant containers, ensure proper ventilation and adopt airtight storage systems to reduce fungal contamination. Agricultural extension services must focus on educating dairy farmers about the risks of fungal contamination and proper feed storage and management practices. Practical training programmes should be implemented to guide farmers on minimizing contamination and improving feed quality. Additionally, local agricultural authorities should establish regular monitoring and surveillance systems for feed quality, particularly in regions with high fungal contamination risks. Early detection programmes using effective methods, such as UV fluorescence and ammonia vapour tests, should be prioritized to minimize contamination and ensure feed safety. Finally, given the influence of temperature and humidity on fungal growth, climate‐adaptive agricultural policies are necessary. These could include developing drought‐resistant crops and alternative feed sources to improve feed security and reduce contamination risks.

## Author Contributions

**Sisay Weldegebriel Zeweld**: conceptualization, methodology, investigation, data curation, formal analysis, writing – original draft, visualization, project administration. **Enquebaher Kassaye Tarekegn**: supervision, validation, writing – review and editing. **Meressa Abraha Welearegay**: supervision, resources, writing – review and editing.

## Ethics Statement

Ethical approval for this study was not required as per the guidelines of the Mekelle University Institutional Review Board, which does not mandate approval for questionnaire‐based studies that do not involve sensitive personal data or pose risks to participants. However, informed consent was obtained from all participants before data collection, and confidentiality was maintained throughout the study in accordance with ethical research principles.

## Conflicts of Interest

The authors declare no conflicts of interest.

### Peer Review

The peer review history for this article is available at https://publons.com/publon/10.1002/vms3.70362.

## Data Availability

Available from the corresponding author upon reasonable request.
